# Symptom Duration and Risk Factors for Delayed Return to Usual Health Among Outpatients with COVID-19 in a Multistate Health Care Systems Network — United States, March–June 2020

**DOI:** 10.15585/mmwr.mm6930e1

**Published:** 2020-07-31

**Authors:** Mark W. Tenforde, Sara S. Kim, Christopher J. Lindsell, Erica Billig Rose, Nathan I. Shapiro, D. Clark Files, Kevin W. Gibbs, Heidi L. Erickson, Jay S. Steingrub, Howard A. Smithline, Michelle N. Gong, Michael S. Aboodi, Matthew C. Exline, Daniel J. Henning, Jennifer G. Wilson, Akram Khan, Nida Qadir, Samuel M. Brown, Ithan D. Peltan, Todd W. Rice, David N. Hager, Adit A. Ginde, William B. Stubblefield, Manish M. Patel, Wesley H. Self, Leora R. Feldstein, IVY Network Investigators, CDC COVID-19 Response Team, IVY Network Investigators, Kimberly W. Hart, Robert McClellan, CDC COVID-19 Response Team, Layne Dorough, Nicole Dzuris, Eric P. Griggs, Ahmed M. Kassem, Paula L. Marcet, Constance E. Ogokeh, Courtney N. Sciarratta, Akshita Siddula, Emily R. Smith, Michael J. Wu

**Affiliations:** ^1^CDC COVID-19 Response Team; ^2^Oak Ridge Institute for Science and Education, Oak Ridge, Tennessee; ^3^Vanderbilt University Medical Center, Nashville, Tennessee; ^4^Beth Israel Deaconess Medical Center, Boston, Massachusetts; ^5^Wake Forest University Baptist Medical Center, Winston-Salem, North Carolina; ^6^Hennepin County Medical Center, Minneapolis, Minnesota; ^7^Baystate Medical Center, Springfield, Massachusetts; ^8^Montefiore Medical Center and Albert Einstein College of Medicine, Bronx, New York; ^9^Ohio State University Wexner Medical Center, Columbus, Ohio; ^10^University of Washington Medical Center, Seattle, Washington; ^11^Stanford University Medical Center, Palo Alto, California; ^12^Oregon Health & Sciences University, Portland, Oregon; ^13^UCLA Medical Center, Los Angeles, California; ^14^Intermountain Healthcare, Salt Lake City, Utah; ^15^Johns Hopkins Hospital, Baltimore, Maryland; ^16^University of Colorado School of Medicine, Aurora, Colorado.; Vanderbilt University Medical Center; Vanderbilt University Medical Center.; CDC COVID-19 Response Team; CDC COVID-19 Response Team; CDC COVID-19 Response Team; CDC COVID-19 Response Team; CDC COVID-19 Response Team; CDC COVID-19 Response Team; CDC COVID-19 Response Team; CDC COVID-19 Response Team; CDC COVID-19 Response Team; CDC COVID-19 Response Team.

Prolonged symptom duration and disability are common in adults hospitalized with severe coronavirus disease 2019 (COVID-19). Characterizing return to baseline health among outpatients with milder COVID-19 illness is important for understanding the full spectrum of COVID-19–associated illness and tailoring public health messaging, interventions, and policy. During April 15–June 25, 2020, telephone interviews were conducted with a random sample of adults aged ≥18 years who had a first positive reverse transcription–polymerase chain reaction (RT-PCR) test for SARS-CoV-2, the virus that causes COVID-19, at an outpatient visit at one of 14 U.S. academic health care systems in 13 states. Interviews were conducted 14–21 days after the test date. Respondents were asked about demographic characteristics, baseline chronic medical conditions, symptoms present at the time of testing, whether those symptoms had resolved by the interview date, and whether they had returned to their usual state of health at the time of interview. Among 292 respondents, 94% (274) reported experiencing one or more symptoms at the time of testing; 35% of these symptomatic respondents reported not having returned to their usual state of health by the date of the interview (median = 16 days from testing date), including 26% among those aged 18–34 years, 32% among those aged 35–49 years, and 47% among those aged ≥50 years. Among respondents reporting cough, fatigue, or shortness of breath at the time of testing, 43%, 35%, and 29%, respectively, continued to experience these symptoms at the time of the interview. These findings indicate that COVID-19 can result in prolonged illness even among persons with milder outpatient illness, including young adults. Effective public health messaging targeting these groups is warranted. Preventative measures, including social distancing, frequent handwashing, and the consistent and correct use of face coverings in public, should be strongly encouraged to slow the spread of SARS-CoV-2.

Prolonged illness is well described in adults with severe COVID-19 requiring hospitalization, especially among older adults (*1*,*2*). Recently, the number of SARS-CoV-2 infections in persons first evaluated as outpatients have increased, including cases among younger adults (*3*). A better understanding of convalescence and symptom duration among outpatients with COVID-19 can help direct care, inform interventions to reduce transmission, and tailor public health messaging.

The Influenza Vaccine Effectiveness in the Critically Ill (IVY) Network, a collaboration of U.S. health care systems, is conducting epidemiologic studies on COVID-19 in both inpatient and outpatient settings (*4*,*5*). Fourteen predominantly urban academic health systems in 13 states each submitted a list of adults with positive SARS-CoV-2 RT-PCR test results obtained during March 31–June 4, 2020, to Vanderbilt University Medical Center. Site-specific random sampling was then performed on a subset of these patients who were tested as outpatients and included patients tested in the emergency department (ED) who were not admitted to the hospital at the testing encounter and those tested in other outpatient clinics. At 14–21 days from the test date, CDC personnel interviewed the randomly sampled patients or their proxies by telephone to obtain self-reported baseline demographic, socioeconomic, and underlying health information, including the presence of chronic medical conditions. Call attempts were made for up to seven consecutive days, and interviews were conducted in several languages (*4*). Respondents were asked to report the number of days they felt unwell before the test date, COVID-19–related symptoms experienced at the time of testing (*6*), whether symptoms had resolved by the date of the interview, and whether the patient had returned to their usual state of health. For this data analysis, respondents were excluded if they did not complete the interview, if a proxy (e.g., family member) completed the interview (because of their incomplete knowledge of symptoms), if they reported a previous positive SARS-CoV-2 test (because the reference date for symptoms questions was unclear), or (because this analysis focused on symptomatic persons) if they did not answer symptoms questions or denied all symptoms at testing.

Descriptive statistics were used to compare characteristics among respondents who reported returning and not returning to their usual state of health by the date of the interview. Generalized estimating equation regression models with exchangeable correlation structure accounting for clustering by site were fitted to evaluate the association between baseline characteristics and return to usual health, adjusting for potential a priori-selected confounders. Resolution and duration of individual symptoms were also assessed. Statistical analyses were conducted using Stata software (version 16; StataCorp).

At least one telephone call was attempted for 582 patients (including 175 [30%] who were tested in an ED and 407 [70%] in non-ED settings), with 325 (56%) interviews completed (89 [27%] ED and 236 [73%] non-ED). Among 257 nonrespondents, 178 could not be reached, 37 requested a callback but could not be reached on further call attempts, 28 refused the interview, and 14 had a language barrier. Among the 325 completed interviews, 31 were excluded: nine (3%) because a proxy was interviewed, 17 (5%) because a previous positive SARS-CoV-2 test was reported, and five (2%) who did not answer the symptoms questions. Two additional respondents were called prematurely at 7 days and were also excluded.* Among the 292 remaining patient respondents, 274 (94%) reported one or more symptoms at testing and were included in this data analysis. Following outpatient testing, 7% (19 of 262 with available data) reported later being hospitalized, a median of 3.5 days after the test date. The median age of symptomatic respondents was 42.5 years (interquartile range [IQR] = 31–54 years), 142 (52%) were female, 98 (36%) were Hispanic, 96 (35%) were non-Hispanic white, 48 (18%) were non-Hispanic black, and 32 (12%) were other non-Hispanic race. Overall, 141 of 264 (53%) with available data reported one or more chronic medical conditions. The median interval from test to interview date was 16 days (IQR = 14–19 days); the median number of days respondents reported feeling unwell before being tested for SARS-CoV-2 was 3 (IQR = 2–7 days).

## Return to Usual State of Health

Among the 270 of 274 interviewees with available data on return to usual health,^†^ 175 (65%) reported that they had returned to their usual state of health a median of 7 days (IQR = 5–12 days) from the date of testing (Table 1). Ninety-five (35%) reported that they had not returned to their usual state of health at the time of interview. The proportion who had not returned to their usual state of health differed across age groups: 26% of interviewees aged 18–34 years, 32% aged 35–49 years, and 47% aged ≥50 years reported not having returned to their usual state of health (p = 0.010) within 14–21 days after receiving a positive test result. Presence of chronic conditions also affected return to health rates; among 180 persons with no or one chronic medical condition, 39 with two chronic medical conditions, and 44 with three or more chronic medical conditions, 28%, 46%, and 57%, respectively, reported not having returned to their usual state of health (p = 0.003) within 14–21 days after having a positive test result. Among respondents aged 18–34 years with no chronic medical condition, 19% (nine of 48) reported not having returned to their usual state of health. Adjusting for other factors, age ≥50 versus 18–34 years (adjusted odds ratio [aOR] = 2.29; 95% confidence interval [CI] = 1.14–4.58) and reporting three or more versus no chronic medical conditions (aOR = 2.29; 95% CI = 1.07–4.90) were associated with not having returned to usual health (Table 2). Obesity (body mass index ≥30 kg per m^2^) (aOR 2.31; 95% CI = 1.21–4.42) and reporting a psychiatric condition^§^ (aOR 2.32; 95% CI = 1.17–4.58) also were associated with more than twofold odds of not returning to the patient’s usual health after adjusting for age, sex, and race/ethnicity.

**TABLE 1:** Characteristics of symptomatic outpatients with SARS-CoV-2 real-time reverse transcription–polymerase chain reaction (RT-PCR)—positive test results (N = 270)* who reported returning to usual state of health or not returning to usual state of health at an interview conducted 14–21 days after testing — 14 academic health care systems,^†^ United States, March–June 2020

Characteristic	Total	Returned to usual health, no. (row %)		P-value^§^
		Yes (n = 175)	No (n = 95)	
**Sex**				0.14
Women	**140**	85 (61)	55 (39)	
Men	**130**	90 (69)	40 (31)	
**Age group (yrs)**				0.010
18–34	**85**	63 (74)	22 (26)	
35–49	**96**	65 (68)	31 (32)	
≥50	**89**	47 (53)	42 (47)	
**Race/Ethnicity**				0.29
White, non-Hispanic	**94**	58 (62)	36 (38)	
Black, non-Hispanic	**46**	26 (57)	20 (43)	
Other race, non-Hispanic	**32**	24 (75)	8 (25)	
Hispanic	**98**	67 (68)	31 (32)	
**Insurance (14 missing)**				0.69
No	**46**	31 (67)	15 (33)	
Yes	**210**	135 (64)	75 (36)	
**No. of medical conditions (7 missing)**				0.003
0	**123**	87 (71)	36 (29)	
1	**57**	41 (72)	16 (28)	
2	**39**	21 (54)	18 (46)	
≥3	**44**	19 (43)	25 (57)	
**Individual medical conditions (7 missing all)** ^¶^				
Hypertension	**64**	33 (52)	31 (48)	0.018
Obesity (body mass index >30 kg/m^2^)	**51**	23 (45)	28 (55)	0.002
Psychiatric condition	**49**	23 (47)	26 (53)	0.007
Asthma	**36**	23 (64)	13 (36)	0.99
Diabetes	**28**	16 (57)	12 (43)	0.43
Immunosuppressive condition	**15**	6 (40)	9 (60)	0.047
Autoimmune condition	**13**	7 (54)	6 (46)	0.44
Blood disorder	**8**	4 (50)	4 (50)	0.47
Chronic kidney disease	**7**	3 (43)	4 (57)	0.26
Chronic obstructive pulmonary disease	**7**	4 (57)	3 (43)	0.71
Liver disease	**6**	4 (67)	2 (33)	1.00
Neurologic condition	**6**	3 (50)	3 (50)	0.48
Coronary artery disease	**4**	3 (75)	1 (25)	1.00
Congestive heart failure	**2**	2 (100)	0 (0)	0.54

* 294 patients responded to an interview 2–3 weeks after testing, did not report a previous positive SARS-CoV-2 test before the reference test, and answered questions about symptoms. Of these, 276 (94%) reported one or more symptoms at the time of SARS-CoV-2 RT-PCR testing, with 272 (99%) reporting whether they had returned to their usual state of health by the time of the interview. Two additional patients excluded who were called at 7 days, with 270 included here.

^†^ Patients were randomly sampled from fourteen academic healthcare systems in 13 states (University of Washington [Washington], Oregon Health and Sciences University [Oregon], University of California Los Angeles and Stanford University [California], Hennepin County Medical Center [Minnesota], Vanderbilt University [Tennessee], Ohio State University [Ohio], Wake Forest University [North Carolina], Montefiore Medical Center [New York], Beth Israel Deaconess Medical Center and Baystate Medical Center [Massachusetts], Intermountain Healthcare [Utah/Idaho], University of Colorado Hospital [Colorado], and Johns Hopkins University [Maryland]).

^§^ Respondents who reported returning to usual health and respondents who reported not returning to usual health were compared using the chi-square test or Fisher's exact test.

^¶ ^Excluding seven (3%) patients who did not answer questions about chronic underlying medical conditions; for those who answered questions about underlying conditions, some respondents were missing data on obesity (two), neurologic conditions (one), and psychiatric conditions (one).

**TABLE 2:** Characteristics associated with not returning to usual health among symptomatic outpatients with SARS-CoV-2 real-time reverse transcription–polymerase chain reaction (RT-PCR)–positive test results (N = 270)* reported at an interview conducted 14–21 days after testing — 14 academic health care systems,^†^ United States, March–June 2020

Characteristic	Odds of not returning to “usual health” at 14–21 days after testing	
	Unadjusted odds ratio (95% CI)^§^	Adjusted odds ratio (95% CI)^§,¶^
**Age group (yrs)**		
18–34	Referent	Referent
35–49	1.40 (0.73–2.67)	1.38 (0.71–2.69)
≥50	2.64 (1.39–5.00)	2.29 (1.14–4.58)
**Sex**		
Women	Referent	Referent
Men	0.68 (0.41–1.13)	0.80 (0.46–1.38)
**Race/Ethnicity**		
White, non-Hispanic	Referent	Referent
Black, non-Hispanic	1.23 (0.60–2.53)	1.13 (0.53–2.45)
Other, non-Hispanic	0.53 (0.21–1.31)	0.63 (0.24–1.61)
Hispanic	0.74 (0.40–1.34)	0.83 (0.44–1.58)
**No. of medical conditions**		
0	Referent	Referent
1	0.94 (0.47–1.89)	0.74 (0.35–1.55)
2	2.09 (1.00–4.38)	1.50 (0.68–3.33)
≥3	3.19 (1.56–6.50)	2.29 (1.07–4.90)
Individual medical conditions**		
Hypertension	1.98 (1.12–3.52)	1.30 (0.67–2.51)
Obesity (BMI >30 kg/m^2^)	2.65 (1.42–4.95)	2.31 (1.21–4.42)
Psychiatric condition	2.42 (1.29–4.56)	2.32 (1.17–4.58)
Asthma	1.00 (0.48–2.08)	1.02 (0.47–2.20)
Diabetes	1.38 (0.62–3.05)	1.06 (0.46–2.44)
Immunosuppressive condition	2.84 (0.98–8.26)	2.33 (0.77–7.04)
Autoimmune condition	1.55 (0.51–4.76)	1.05 (0.32–3.46)
Blood disorder	1.82 (0.45–7.45)	1.43 (0.33–6.24)
Chronic kidney disease	2.42 (0.53–11.05)	2.36 (0.48–11.51)
Chronic obstructive pulmonary disease	1.34 (0.29–6.12)	0.70 (0.14–3.48)
Liver disease	0.88 (0.16–4.90)	0.72 (0.12–4.25)
Neurologic condition	1.78 (0.35–9.01)	1.23 (0.23–6.62)
Coronary artery disease	0.58 (0.06–5.70)	0.48 (0.05–4.92)
Congestive heart failure	—	—

**Abbreviations**: BMI = body mass index; CI = confidence interval.

* 294 patients responded to 14–21-day interview, did not report a previous positive SARS-CoV-2 test before the reference test, and answered questions about symptoms; 276 (94%) of these reported one or more symptoms at the time of SARS-CoV-2 RT-PCR testing, with 272 (99%) reporting whether they had returned to their usual state of health by the time of the interview. Two additional patients who were called at 7 days were excluded, with 270 included here.

^†^ Patients were randomly sampled from academic healthcare systems in 13 states (University of Washington [Washington], Oregon Health and Sciences University [Oregon], University of California Los Angeles and Stanford University [California], Hennepin County Medical Center [Minnesota], Vanderbilt University [Tennessee], Ohio State University [Ohio], Wake Forest University [North Carolina], Montefiore Medical Center [New York], Beth Israel Deaconess Medical Center and Baystate Medical Center [Massachusetts], Intermountain Healthcare [Utah/Idaho], University of Colorado Hospital [Colorado], and Johns Hopkins University [Maryland]).

^§^ For this analysis, generalized estimation equation (GEE) models with exchangeable correlation structure were used to estimate the association between characteristics and the odds of not returning to usual health by the date of the 14–21-day interview. GEE models were used to account for clustering of cases by site. 95% CIs including 1.00 are not considered statistically significant.

^¶^ In adjusted GEE models for age, sex, race/ethnicity, and number of chronic medical conditions, the other variables were used to adjust for potential confounders. Models for individual conditions (e.g., hypertension) were adjusted for age, sex, and race/ethnicity.

** Medical conditions are not exclusive and individual patients could have more than one chronic medical condition.

## Resolution of Symptoms and Duration

Among the 274 symptomatic outpatients, the median number of symptoms was seven of 17 listed in the interview tool (IQR = 5–10), with fatigue (71%), cough (61%), and headache (61%) those most commonly reported (Figure). Among respondents who reported fever and chills on the day of testing, these resolved in 97% and 96% of respondents, respectively. Symptoms least likely to have resolved included cough (not resolved in 43% [71 of 166]) and fatigue (not resolved in 35% [68 of 192]); among 90 who reported shortness of breath at the time of testing, this symptom had not resolved in 26 (29%). The median interval to symptom resolution among those who reported individual symptoms at the time of testing but not at the time of the interview ranged from 4 to 8 days from the test date, with the longest intervals reported for loss of smell (median = 8 days; IQR = 5–10.5 days) and loss of taste (median = 8 days; IQR = 4–10 days). Among respondents who reported returning to their usual state of health, 34% (59 of 175) still reported one or more of the 17 queried COVID-related symptoms at the time of the interview.

**FIGURE:**
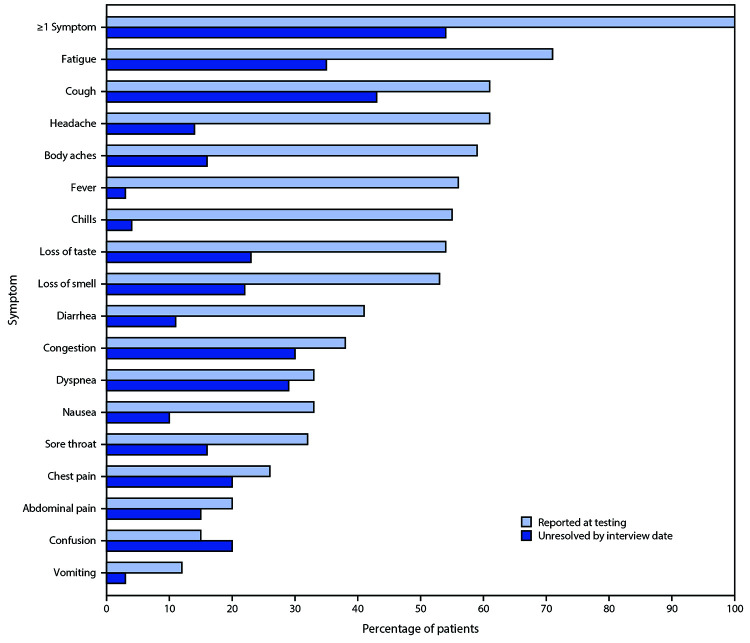
Self-reported symptoms at the time of positive SARS-CoV-2 reverse transcription–polymerase chain reaction (RT-PCR) testing results and unresolved symptoms 14–21 days later among outpatients (N = 274)* — 14 academic health care systems,^†^ United States, March–June 2020 * 294 patients responded to 14–21-day interview, did not report a previous positive SARS-CoV-2 test before the reference test, and answered questions about symptoms; 276 (94%) of these reported one or more symptoms at the time of SARS-CoV-2 RT-PCR testing; those who were interviewed at 7 days were excluded, with 274 included here. ^†^ Patients were randomly sampled from 14 academic health care systems in 13 states (University of Washington [Washington], Oregon Health and Sciences University [Oregon], University of California Los Angeles and Stanford University [California], Hennepin County Medical Center [Minnesota], Vanderbilt University [Tennessee], Ohio State University [Ohio], Wake Forest University [North Carolina], Montefiore Medical Center [New York], Beth Israel Deaconess Medical Center and Baystate Medical Center [Massachusetts], Intermountain Healthcare [Utah/Idaho], University of Colorado Hospital [Colorado], and Johns Hopkins University [Maryland]).

## Discussion

Most studies to date have focused on symptoms duration and clinical outcomes in adults hospitalized with severe COVID-19 (*1*,*2*). This report indicates that even among symptomatic adults tested in outpatient settings, it might take weeks for resolution of symptoms and return to usual health. Not returning to usual health within 2–3 weeks of testing was reported by approximately one third of respondents. Even among young adults aged 18–34 years with no chronic medical conditions, nearly one in five reported that they had not returned to their usual state of health 14–21 days after testing. In contrast, over 90% of outpatients with influenza recover within approximately 2 weeks of having a positive test result (*7*). Older age and presence of multiple chronic medical conditions have previously been associated with illness severity among adults hospitalized with COVID-19 (*8*,*9*); in this study, both were also associated with prolonged illness in an outpatient population. Whereas previous studies have found race/ethnicity to be a risk factor for severe COVID-19 illness (*10*), this study of patients whose illness was diagnosed in an outpatient setting did not find an association between race/ethnicity and return to usual health although the modest number of respondents might have limited our ability to detect associations. The finding of an association between chronic psychiatric conditions and delayed return to usual health requires further evaluation. These findings have important implications for understanding the full effects of COVID-19, even in persons with milder outpatient illness. Notably, convalescence can be prolonged even in young adults without chronic medical conditions, potentially leading to prolonged absence from work, studies, or other activities.

The findings in this report are subject to at least three limitations. First, nonrespondents might have differed from survey respondents; for example, those with more severe illness might have been less likely to respond to telephone calls if they were subsequently hospitalized and unable to answer the telephone. Second, symptoms that resolved before the test date or that commenced after the date of testing were not recorded in this survey. Finally, as a telephone survey, this study relied on patient self-report and might have been subject to incomplete recall or recall bias.

Nonhospitalized COVID-19 illness can result in prolonged illness and persistent symptoms, even in young adults and persons with no or few chronic underlying medical conditions. Public health messaging should target populations that might not perceive COVID-19 illness as being severe or prolonged, including young adults and those without chronic underlying medical conditions. Preventative measures, including social distancing, frequent handwashing, and the consistent and correct use of face coverings in public, should be strongly encouraged to slow the spread of SARS-CoV-2.

**Summary**

**What is already known about this topic?**

Relatively little is known about the clinical course of COVID-19 and return to baseline health for persons with milder, outpatient illness.

**What is added by this report?**

In a multistate telephone survey of symptomatic adults who had a positive outpatient test result for SARS-CoV-2 infection, 35% had not returned to their usual state of health when interviewed 2–3 weeks after testing. Among persons aged 18–34 years with no chronic medical conditions, one in five had not returned to their usual state of health.

**What are the implications for public health practice?**

COVID-19 can result in prolonged illness, even among young adults without underlying chronic medical conditions. Effective public health messaging targeting these groups is warranted.

## Footnotes

* Two patients interviewed early at 12 days and three interviewed at 13 days after testing were included. Two patients who requested interview after 21 days because they were unavailable at 14–21 days were included (interviews were conducted at 25 and 26 days). All other included respondents were interviewed 14–21 days after testing.

† Patients were asked the question “Would you say that you are feeling back to your usual health?”

§ Psychiatric conditions included anxiety disorder (38), depression (21), posttraumatic stress disorder (two), paranoia (two), obsessive-compulsive disorder (one), schizophrenia (one); some patients reported more than one condition.
